# Antiviral therapies against Ebola and other emerging viral diseases using existing medicines that block virus entry

**DOI:** 10.12688/f1000research.6085.2

**Published:** 2015-02-10

**Authors:** Jason Long, Edward Wright, Eleonora Molesti, Nigel Temperton, Wendy Barclay

**Affiliations:** 1 Section of Virology, St Mary’s Campus, Imperial College London, London, W2 1PG, UK; 2 Viral Pseudotype Unit (Fitzrovia), Faculty of Science and Technology, University of Westminster, London, W1W 6UW, UK; 3 Viral Pseudotype Unit, School of Pharmacy, University of Kent, Chatham Maritime, Kent, ME4 4TB, UK

**Keywords:** ebola, emerging viral disease, avian influenze, H5N1, Marburg, chloroquine, omemprazole, esomeprazole

## Abstract

Emerging viral diseases pose a threat to the global population as intervention strategies are mainly limited to basic containment due to the lack of efficacious and approved vaccines and antiviral drugs. The former was the only available intervention when the current unprecedented Ebolavirus (EBOV) outbreak in West Africa began. Prior to this, the development of EBOV vaccines and anti-viral therapies required time and resources that were not available. Therefore, focus has turned to re-purposing of existing, licenced medicines that may limit the morbidity and mortality rates of EBOV and could be used immediately. Here we test three such medicines and measure their ability to inhibit pseudotype viruses (PVs) of two EBOV species, Marburg virus (MARV) and avian influenza H5 (FLU-H5). We confirm the ability of chloroquine (CQ) to inhibit viral entry in a pH specific manner. The commonly used proton pump inhibitors, Omeprazole and Esomeprazole were also able to inhibit entry of all PVs tested but at higher drug concentrations than may be achieved *in vivo*. We propose CQ as a priority candidate to consider for treatment of EBOV.

## Introduction

Emerging pathogens such as Ebolaviruses (EBOV), Avian Influenza viruses, Severe Acute Respiratory Syndrome (SARS) virus, Middle-East coronavirus (MERS), Chikungunya virus (CHIKV) and Dengue virus pose public health challenges that demand researchers and governments work together to assess their pandemic potential and plan mitigating strategies. Of the five species of EBOV belonging to the *Filoviridae* (including *Zaire ebolavirus* (EBOV-Z), *Bundibugyo ebolavirus* (EBOV-B), *Reston ebolavirus*, *Sudan ebolavirus* (EBOV-S) and *Tai Forest ebolavirus* ^1^), EBOV-Z and EBOV-S are responsible for the majority of outbreaks of highly pathogenic haemorrhagic fevers causing high fatality rates ^2^. Past outbreaks have been of limited size affecting a local population, however a strain of EBOV-Z is the causative agent of the current outbreak that began in late 2013 and has since become an unprecedented and devastating epidemic ^3, 4^, resulting in over 20,000 suspected cases, of which those confirmed had a case fatality rate of around 60% in the afflicted West African countries ( http://apps.who.int/gho/data/view.ebola-sitrep.ebola-summary-20150107?lang=en and http://www.who.int/csr/disease/ebola/situation-reports/en/). Towards the end of 2014 the trend in case numbers reversed in Liberia and the epidemic slowed in Sierra Leone and Guinea, but the virus continues to transit in new geographical areas ^5^. This epidemic has triggered a significant global health response relying on primary hygiene and other containment measures that have proved successful in limiting the spread of the virus in previous outbreaks. Given the scale of this outbreak and the fear that traditional containment measures may fail to prevent global spread, several vaccines have been fast-tracked into phase I clinical trials ^6– 8^ although even if proved efficacious, the limited supply of sufficient quantities of vaccine will hinder their use in the current situation. For disease treatment, patients suffering a haemorrhagic fever have relied on the clinical management of symptoms ( http://www.cdc.gov/vhf/ebola/treatment/), with a handful of patients in this outbreak receiving experimental therapies such as ZMapp, TKM-Ebola, brincidofovir and favipiravir ( http://www.nature.com/news/ebola-trials-to-start-in-december-1.16342) ^9– 12^. Alternatively antibody treatment by transfusion therapy using blood or plasma from Ebola virus survivors has been approved ^11, 13– 16^; although issues with safety and lack of resources for this method limit its suitability in West Africa today. Having no approved or widely available therapeutics for EBOV, as with many other emerging viral diseases, focus has turned to possible re-purposing of drugs already licensed for other uses by the EMA and FDA. Several clinically approved drugs have been identified by researchers ^17– 20^, including amiodarone, one of the several cationic amphiphiles found to inhibit filovirus entry which is currently being trialled in Sierra Leone ^21^. However reservations have been expressed about the complications that could be caused by side effects of the drug in EBOV patients. The anti-malarial drug chloroquine (CQ) has also been shown to inhibit EBOV entry and protected mice from EBOV infection ^18, 22^ and has been previously highlighted as a possible drug to treat EBOV infection ^11^.

The possible difficulties that may arise with use of re-purposed drugs include unforeseen interactions between virus/drug and host causing exacerbation of disease. Therefore it is important to try and understand the mechanism of virus inhibition by such drugs. To this end we re-examined the anti-viral properties of CQ, and show here that it inhibited the pH-dependent endosomal entry of a pseudotyped virus (PV) bearing EBOV glycoproteins, in the same way as did the potent and specific vacuolar-ATPase (vATPase) inhibitor bafilyomycin A1 (BafA1) (a non-medical laboratory compound). We also show that licensed and widely used proton pump inhibitors (PPIs) for treatment of gastric acid reflux, omeprazole (OM) and esomeprazole (ESOM), inhibited PV EBOV entry, likely by their off-target inhibitory activity on endosomal vATPase.

## Methods

### Cell culture

Human embryonic kidney (293T/17) (ATCC) and Human lung adenocarcinoma epithelial cells (A549) (ATTC) were maintained in Dulbecco’s modified Eagle’s medium (DMEM; Invitrogen) supplemented with 10% fetal calf serum (FCS) (Biosera) and 1% Penicillin-streptomycin (PS) (Invitrogen). The cell lines were maintained at 37°C in a 5% CO _2_ atmosphere.

### Compounds

Chloroquine diphosphate salt (CQ), bafilomycin A1 from *Streptomyces griseus* (BafA1), omeprazole (OM) and esomeprazole magnesium hydrate (ESOM) (Sigma) were resuspended as per manufacturer’s instructions and aliquots stored at -20°C: CQ was prepared in sterile dH _2_O; BafA1, OM and ESOM were prepared in sterile DMSO (Sigma).

### Plasmid constructs

The Bundibugyo ebolavirus (EBOV-B) envelope glycoprotein (GP) (FJ217161) coding sequence was synthesised (Bio Basic Inc.) and the HA glycoprotein of avian influenza A/turkey/England/50-92/91(H5N1) (FLU-H5) was amplified from the HA plasmid of the H5N1 reverse genetics system ^23^. Both were sub-cloned into the pCAGGS expression vector. Expression vectors containing the envelope glycoproteins of Zaire Ebolavirus (Mayinga) (EBOV_Z), Marburg virus (Lake Victoria isolate; MARV) and Gibbon Ape Leukemia Virus (GALV) (modified to contain the trans-membrane domain of amphotropic murine leukemia virus (A-MLV) envelope glycoprotein) are described previously ^24, 25^. The *Renilla* luciferase gene was sub-cloned into pCAGGS expressing vector from a minigenome reporter described previously ^26^.

### Generation of pseudotype viruses

The generation of all lentiviral pseudotype viruses was based on the methods detailed previously ^27– 29^. Briefly, 293T/17 cells were seeded into 10cm ^3^ tissue culture plates (Nunc™ Thermo Scientific). The HIV gag-pol plasmid, pCMV-Δ8.91 and the firefly luciferase reporter construct, pCSFLW, were transfected together with either influenza HA, GALV, EBOV or Marburg GP expression constructs at a ratio of 1:1.5:1 (core:reporter:envelope) using Fugene6 transfection reagent (Promega). At 24 h post-transfection, cells were washed and fresh media applied. For the generation of H5 PVs, 1U exogenous recombinant neuraminidase from *Clostridium perfringens* (Sigma-Aldrich) was also added 24 h after transfection to effect egress from the producer cells. PV supernatants were harvested at 48 and 72 h post-transfection and passed through a 0.45m pore filter. EBOV PVs were aliquoted and stored at 4°C; the remaining PVs were stored at -80°C.

### Entry inhibition assay

293T cells in 10cm ^3^ plates were transfected with 15ug of *Renilla* luciferase expressing plasmid using Lipofectamine 2000 according to manufacturer’s instructions (Life Technologies™). CQ, BafA1, OM and ESOM were serially diluted in 96-well white-bottomed plates (Nunc™ Thermo Scientific) to give the final described concentrations. After 20h the transfected cells were trypsinised and 1×10 ^4^ cells were added to each well. After 30min cells were transduced with no more than 1×10 ^5^ RLU of PV per well (estimated from raw RLU values of previously infected 293T cells), and to an equal volume per well. 48 h later supernatant was removed and cells were lysed with 30µl of passive lysis buffer (Promega), and firefly/ *Renilla* luciferase activity measured using a FLUOstar Omega plate reader (BMG Labtech) and the Dual luciferase assay system (Promega).

### Measurement of intracellular pH

A549 cells were pre-treated with drug 1 h before 75nM of the pH sensitive Lysotracker ^®^ Red DND-99 (Life Technologies™) was added to the media of each well ^30^. After 30minutes in growth conditions, cells were analyzed for fluorescence using an Axiovert 40 confocal laser (CFL) microscope and an AxioCam MRc camera (Carl Zeiss).

### Statistical analysis

PV transduction RLUs were normalised to the *Renilla* value in the corresponding wells. Percent infection of each drug dilution was calculated compared to untreated cells. Two-way ANOVA with Bonferroni’s multiple comparisons test between untreated and treated mean values (α-0.05) was performed to measure statistically significant differences. IC _50_ values were calculated using non-linear regression analysis (log[inhibitor] vs normalised response). All manipulation of data was performed on GraphPad Prism 6 (GraphPad software).

## Results

### Inhibition of pseudotype virus entry by exisiting FDA-approved drugs

The envelope glycoproteins of several emerging viruses with high pathogenicity and pandemic potential were used to create lentiviral based pseudotype particles as previously described ^29^. PVs were generated bearing the envelope glycoproteins from Zaire ebolavirus (Mayinga strain) (EBOV-Z), Bundibugyo ebolavirus (EBOV-B), Marburg (Lake Victoria isolate) virus (MARV), H5 HA from a highly pathogenic avian influenza virus A/turkey/England/50-92/91(H5N1) (FLU-H5), and Gibbon Ape Leukaemia virus (GALV). GALV PVs were included because GALV is a virus that does not require acidification of endosomes for its entry into cells. All the PVs generated were shown to transduce 293T cells and firefly luciferase expression from the packaged reporter gene was measured above mock infected cells (non-transduced cells) ( Dataset 1).

In order to assess the ability of CQ, BafA1, OM and ESOM to inhibit PV entry, drugs were serially diluted in triplicate in white bottomed 96-well plates. Next, 293T cells transfected 24 hours previously with a *Renilla* luciferase expression plasmid to allow monitoring of cell viability, were added to each well. Appropriately diluted PVs were then added to each dilution, including a no-drug control. After 48 hours incubation, the supernatant was removed and firefly and *Renilla* luciferase RLUs were recorded using the Dual Luciferase Assay System (Promega).

**Figure 1.**
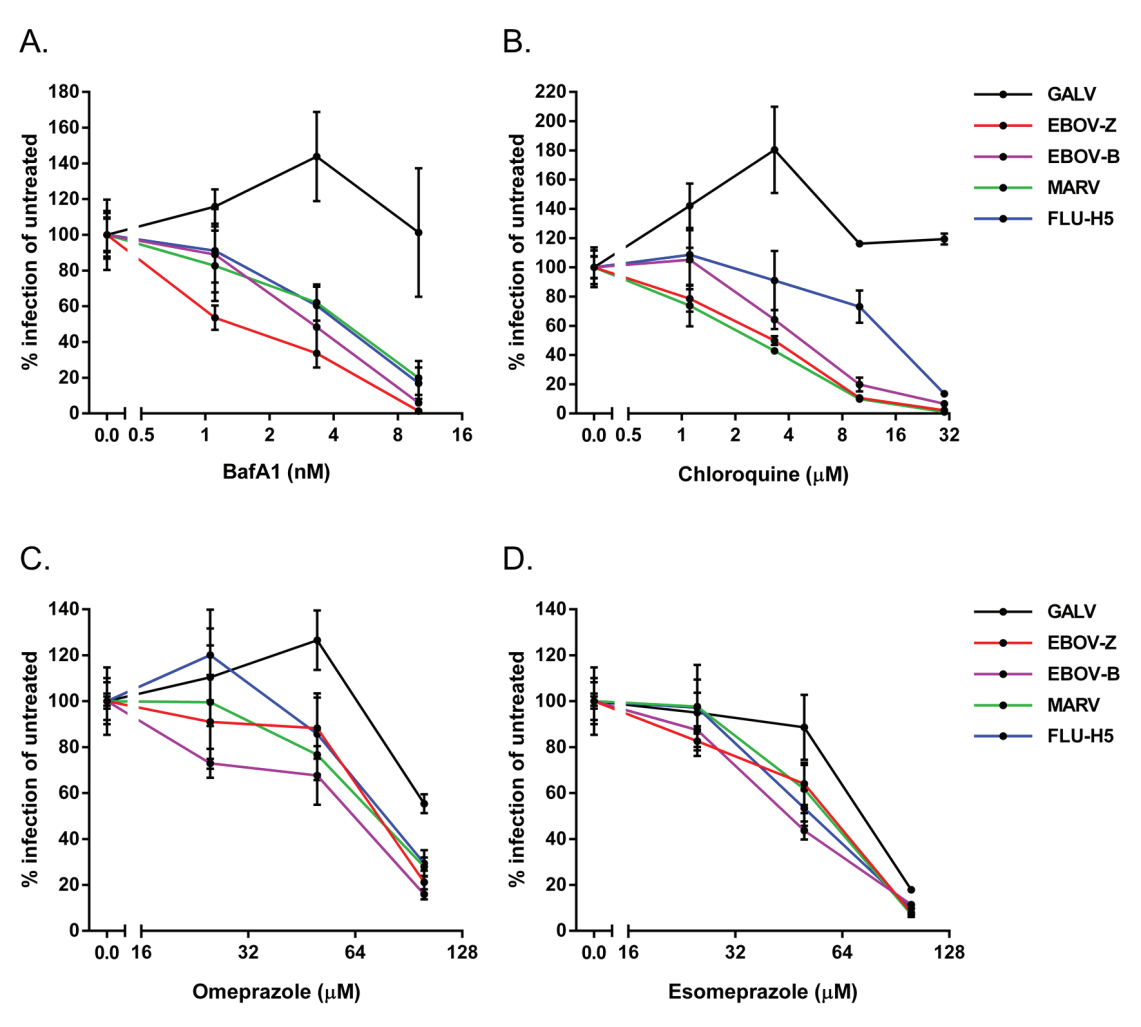
Inhibition of pseudotype virus entry by existing FDA-approved drugs 293T cells previously transfected with a *Renilla* expression plasmid were treated with differing concentrations of drug before being transduced with PV (carried out in triplicate). Data are the percent of infection compared to untreated cells. EBOV-Z, EBOV-B, MARV, FLU-H5 and GALV inhibition was measured for each drug compound. Cells were harvested and firefly and *Renilla* activity measured after 48 h incubation. **A**. Cells were treated with 10, 3.33 and 1.11nM of BafA1. **B**. Cells were treated with 30, 10, 3.33 and 1.11µM of CQ. **C** and **D**. Cells were treated with 100, 50 and 25µM of OM and ESOM, respectively. Statistical analysis of these data are shown in Table 1.

PV RLUs were normalised to the corresponding *Renilla* values, which reduced the edge effect observed in the 96-well plates, and controlled for toxicity of the drugs. Only BafA1 appeared to reduce expression of *Renilla* at the highest concentrations, suggesting cellular toxicity, ( Dataset 1) and visible cytopathic effect was not observed in cells treated by CQ, OM and ESOM at the concentrations used in Figure 1.

**Table 1.** Inhibition of pseudotype viruses by existing FDA-approved drugs.

**BafA1**						
Pseudotype virus	IC _50_ (nM) ^a^	Std. Err.	Significance at dose (nM) (vs. untreated) ^b^			
			1.11	3.33	10	
EBOV-Z	1.213	0.195	ns	*	****	
EBOV-B	3.297	0.233	ns	ns	***	
FLU-H5	3.510	0.282	ns	ns	**	
GALV	ns		ns	ns	ns	
**Chloroquine**						
Pseudotype virus	IC _50_ (μM) ^a^	Std. Err.	Significance at dose (μM) (vs. untreated) ^b^			
			1.11	3.33	10	30
EBOV-Z	3.319	0.147	ns	*	****	****
EBOV-B	3.585	0.198	ns	ns	****	****
MARV	3.192	0.186	ns	**	****	****
FLU-H5	10.44	0.245	ns	ns	ns	****
GALV	ns		ns	****	ns	ns
**Omeprazole**						
Pseudotype virus	IC _50_ (μM) ^a^	Std. Err.	Significance at dose (μM) (vs. untreated) ^b^			
			**25**	**50**	**100**	
EBOV-Z	ns		ns	ns	***	
EBOV-B	50.32	0.234	ns	ns	***	
MARV	52.21	12.290	ns	ns	***	
FLU-H5	50.78	0.562	ns	ns	**	
GALV	ns		ns	ns	ns	
**Esomeprazole**						
Pseudotype virus	IC _50_ (μM) ^a^	Std. Err.	Significance at dose (μM) (vs. untreated) ^b^			
			**25**	**50**	**100**	
EBOV-Z	50.25	0.163	ns	*	****	
EBOV-B	49.89	0.127	ns	***	****	
MARV	50.21	0.174	ns	*	****	
FLU-H5	50.06	0.160	ns	**	****	
GALV	ns		ns	ns	****	

^a^ IC _50_ values were calculated using non-linear regression analysis (log[inhibitor] vs normalised response)

^b^ Two-way ANOVA with Bonferroni’s multiple comparisons test between untreated and treated mean values (α-0.05)

ns P>0.05, * P≤0.05, ** P≤0.01, *** P≤0.001, **** P≤0.0001

Both BafA1 and CQ reduced EBOV-Z, EBOV-B, MARV and FLU-H5 entry in a dose dependent manner ( Figure 1A and B). The IC _50_ value of BafA1 was in the nM range for EBOV-Z, EBOV-B, FLU-H5 and MARV and inhibition of entry was statistically significant at the 10nM concentration compared to the untreated control ( Table 1). CQ inhibited EBOV-Z, EBOV-B, MARV and FLU-H5 with IC _50_ of 3.319, 3.585, 3.192 and 10.44µM respectively, and inhibition was statistically significant ( Table 1). In contrast, GALV entry was augmented by both BafA1 and CQ above that of the untreated cells to a maximum of 143.83% (3.33nM) and 180.38% (3.33µM) respectively. Both OM and ESOM reduced entry of all PVs tested at 100µM but GALV PV was the least affected ( Figure 1C and D). Inhibition of entry for EBOV-Z, EBOV-B, MARV and FLU-H5 PVs by ESOM was significant at 50µM, and GALV PV was not significantly inhibited at this dose ( Figure 1D and Table 1).

### Increasing endosome pH as a mechanism of inhibiting virus pH-dependent entry

**Figure 2.**
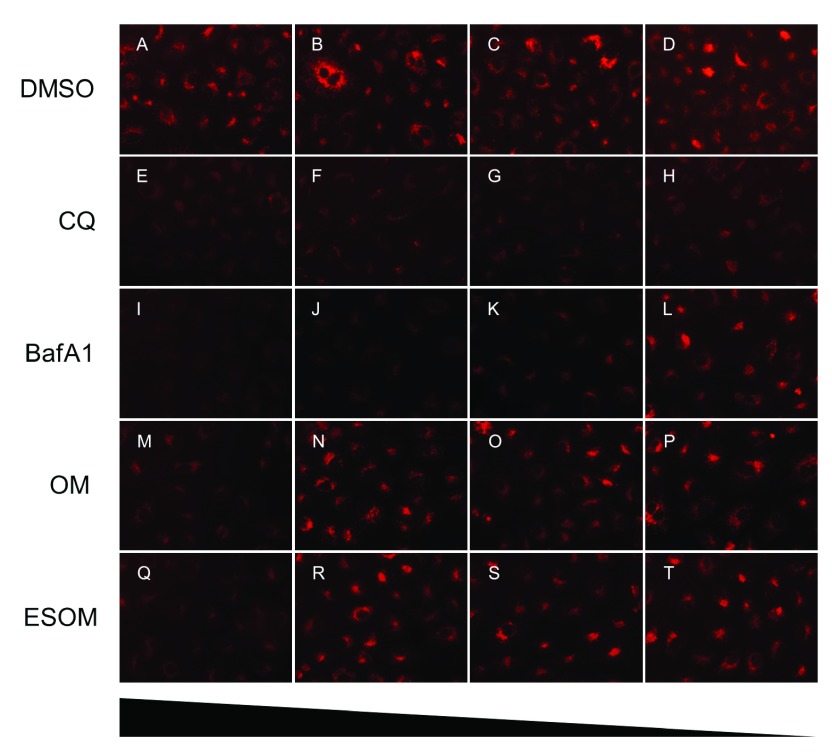
Correlation of decreased pH with inhibitory effect on entry. A549 cells were treated with drug for 1 h before 75nM Lysotracker ^®^ Red DND-99 was added to each well. DMSO (drug vehicle) only was diluted at 30mM, 3mM, 0.3mM and 0.03mM. ( **A–D**). CQ was diluted 30, 10, 3.33 and 1.11µM ( **E–H**), BafA1 was diluted to 10, 3.33 and 1.11nM ( **I–L**) and OM and ESOM were diluted to 100, 50 and 25µM ( **M–P**) and ( **Q–T**) respectively. The level of fluorescence was imaged by confocal microscopy (x50 magnification).

BafA1 and CQ are known endosomal acidification inhibitors (BafA1 being a potent and specific vATPase inhibitor and CQ a licensed lysotropic agent) ^31^. The effects of OM and ESOM on endosomal acidification have also been previously reported ^32, 33^. To confirm that endosomal pH was being affected at doses used here, A549 cells were treated with drug for 1 hour before applying LysoTracker ^®^ Red DND-99 (LifeTechnologies). A549 cells were chosen here because 293T cells are poorly imaged due to their morphology. The lysotracker probe specifically fluoresces in acidic organelles. Fluorescence was decreased in cells treated with BafA1 and CQ in a dose dependant manner, but was unaffected in cells treated with vehicle alone ( Figure 2). OM and ESOM appeared to decrease fluorescence, and therefore increase endosomal pH, only at a concentration of 200µM, higher than that required to inhibit PV entry. Moreover cellular toxicity was observed at this concentration after 24 hours.

Inhibition of pseudotype virus entry by existing FDA-approved drugs 293T cells were transfected 24hours previous then mixed with set dilutions of drug and infected with PVs (carried out in triplicate). After 48hours incubation firefly and Renilla values were measured using FLUOstar Omega plate reader (BMG Labtech). Firelfly values were normalised to the corresponding Renilla value. Percent infection at each dilution was calculated using the average values of the untreated control. *values for both ESOM and OM were shared and performed on the same 96-well plate.

## Conclusions and discussion

After attachment to cells, viruses require a mechanism of fusion to deliver the viral genome. Preventing this action by fusion inhibitors has been successful approach for HIV antiviral therapy ^34^. Unlike HIV, EBOV and many other viruses are dependent on the naturally low pH of acidic endosomes to activate and trigger fusion by their envelope glycoproteins. In this instance, a ‘fusion inhibitor’ could target the host cell machinery preventing acidification of the endosome, working to inhibit virus entry of several different viruses. Here we have reiterated that cell entry by PVs representing EBOV, FLU-H5 and MARV can be inhibited by increasing the endosome pH using BafA1 and CQ ( Figure 1), and this correlates with their ability to prevent the acidification of intracellular organelles ( Figure 2).

CQ has shown antiviral activity against several viruses *in vitro*, including EBOV, influenza, Nipah, Hendra, Dengue and CHIKV ^35– 37^. Disappointingly, this antiviral activity has not always translated into efficacy *in vivo* models or clinical trials, although CQ was effective in a mouse model against EBOV ^18, 35, 38– 42^. The variability in *in vivo* results may depend on study design and strains of virus used. In one study BafA1 treated mice were not protected from influenza infection but treatment with a related compound, SaliPhe, was protective, even though both drugs were potent *in vitro* ^43^. Inhibition of endosome acidification as a target for inhibiting EBOV can be justified by the knowledge that the filoviruses depend on the low pH for two separate steps of their entry pathway. Not only is the fusion by G protein triggered by low pH, but its cleavage into a fusogenic form is carried out by endosomal enzymes cathepsins B and L whose activation is also pH dependent ^44^. Some have argued that G protein cleavage by cathepsin is less essential than previously thought ^45, 46^ and that EBOV species other than Zaire together with closely related MARV do not require cathepsin cleavage for entry ^47, 48^. Nonetheless, entry of MARV PVs was still inhibited in our assays suggesting that inhibiting fusion alone is sufficient.

Recently, using computational modelling, Ekins *et al.* suggested the anti-EBOV mechanism of CQ may be by binding the VP35 protein of EBOV ^49^. If this drug had activity on several steps of the replication cycle it may not only be more effective *in vivo* but it may be even less likely that the virus could mutate to escape inhibition.

At first we were surprised that CQ actually increased entry of GALV PV ( Figure 1). However this effect has been noted before for other retroviruses, including A-MLV and HIV-1, and is accounted for by the inhibitory effect of CQ on the autophagy pathway. CQ prevents degradation of phagosomes that contain virus particles and prevents them from otherwise being degraded ^50– 52^.

CQ has been used for many years as an anti-malarial drug, although it is now only effective in parts of central America and the Caribbean due to accumulation of drug resistance by the plasmodium parasite ^53^. Interestingly, compounds belonging to the omeprazole family have also been described as having anti-malarial properties *in vitro*, possibly via their reported ability to target vATPase in the plasma membrane of Plasmodium parasite ^54^. Soon after its discovery OM was found to also inhibit intracellular vATPase at µM concentrations as opposed to its licensed target of gastric H+/K+-ATPase against which it is effective at much lower concentrations ^32, 33^. Indeed there are a plethora of publications indicating use of OM and ESOM in cancer therapy, as a means to inhibit the characteristic acidic intracellular environment, and thus permit sensitivity to cytotoxic therapies ^55– 59^. A role of OM and ESOM has also been noted in the suppression of bone resorption, another physiological process dependent on pH ^60– 62^. Given the volume of research suggesting these off target effects depend on an ability to affect intracellular pH, we hypothesised that these drugs would, like CQ and BafA1, inhibit EBOV, MARV and influenza virus pH dependent entry. We used GALV as a control again since its entry is reportedly independent of pH. Indeed, EBOV, FLU-H5 and MARV were inhibited by lower doses of OM or ESOM than GALV ( Figure 1 and Table 1). GALV entry was also inhibited at the highest concentration, but we cannot exclude that this was due to a toxic effect that was not measured by the *Renilla* control we employed here. We did not observe as close a correlation between drug doses that mediated the inhibition of EBOV or influenza PV entry and increase in pH of intracellular vesicles for OM and ESOM as for CQ and BafA, ( Figure 1 and Figure 2). More recently, it has been reported that OM and ESOM altered the localisation of vATPase in the cell as well as the pH of intracellular vesicles ^46^ and this may explain their ability to inhibit PV entry more potently than the pH changes we observed would suggest.

Inhibition of influenza virus entry to cells by means of inhibiting acidification of endosomes has been known for decades ^63^, although no current antivirals for influenza have been licensed on this basis. Some epidemiological evidence from population studies suggests that OM could exert a protective effect against influenza-like-illness ^64^, but our studies suggest that doses required for potent inhibition might be difficult to achieve without significant toxicity. Despite these drugs being readily available, even without prescription in some countries, the licensed dosing would generate a plasma concentration reportedly 1.59–9.61µM for ESOM that falls short of the IC _50_ calculated in this study, although higher doses have been used clinically ^65^. Therefore it seems unlikely that OM and ESOM would be a suitable therapy for ebolavirus infection, but more specifically designed vATPase inhibitors may have potential as broad acting antivirals against several emerging viruses in the future. With regard to CQ, the evidence suggests a more promising position for use against ebolavirus. Standard adult dosing (25mg/kg) achieves plasma concentration of 2µM, close to our IC _50_ value against EBOV PV entry. Protection in the mouse model was previously shown with a 90mg/kg dosage ^18, 66^.

Using re-purposed drugs to treat outbreaks of emerging diseases must surely be approached with caution. In Ebola patients with severe life-threatening disease it would be important to ensure that any side effects of a therapy did not enhance disease progression, particularly if higher doses of re-purposed drugs, as suggested here, were considered. On the other hand, CQ has been taken prophylactically in a tropical setting for many years to prevent malaria and we suggest that, with little additional need for scale up of production of a new agent, this might represent a useful adjunct to the current antiviral strategies being trialled in West Africa. We envisage that in contacts of EBOV cases, CQ might decrease the viral load that establishes in the early days after virus transmission. Further work in *in vivo* models including guinea pig and primates should inform about doses and administration regimens.

## Data availability

*Figshare:* Inhibition of pseudotype virus entry by existing FDA-approved drugs. doi: http://dx.doi.org/10.6084/m9.figshare.1294801 ^67^

## References

[1] Li YH Chen SP: Evolutionary history of Ebola virus. *Epidemiol Infect.* 2014;142(6):1138–45. 10.1017/S0950268813002215 24040779 PMC9151191

[2] Feldmann H Geisbert TW: Ebola haemorrhagic fever. *Lancet.* 2011;377(9768):849–62. 10.1016/S0140-6736(10)60667-8 21084112 PMC3406178

[3] Baize S Pannetier D Oestereich L: Emergence of Zaire Ebola virus disease in Guinea. *N Engl J Med.* 2014;371(15):1418–25. 10.1056/NEJMoa1404505 24738640

[4] Gatherer D: The 2014 Ebola virus disease outbreak in West Africa. *J Gen Virol.* 2014;95(Pt 8):1619–24. 10.1099/vir.0.067199-0 24795448

[5] WHO Ebola Response Team: West African Ebola Epidemic after One Year - Slowing but Not Yet under Control. *N Engl J Med.* 2014. 10.1056/NEJMc1414992 25539446 PMC4368109

[6] Kanapathipillai R Henao Restrepo AM Fast P: Ebola vaccine--an urgent international priority. *N Engl J Med.* 2014;371(24):2249–51. 10.1056/NEJMp1412166 25289888

[7] Kibuuka H Berkowitz NM Millard M: Safety and immunogenicity of Ebola virus and Marburg virus glycoprotein DNA vaccines assessed separately and concomitantly in healthy Ugandan adults: a phase 1b, randomised, double-blind, placebo-controlled clinical trial. *Lancet.* 2014;S0140-6736(14)62385-0. 10.1016/S0140-6736(14)62385-0 25540891

[8] Ledgerwood JE DeZure AD Stanley DA: Chimpanzee Adenovirus Vector Ebola Vaccine - Preliminary Report. *N Engl J Med.* 2014. 10.1056/NEJMoa1410863 26287857

[9] Smither SJ Eastaugh LS Steward JA: Post-exposure efficacy of oral T-705 (Favipiravir) against inhalational Ebola virus infection in a mouse model. *Antiviral Res.* 2014;104:153–5. 10.1016/j.antiviral.2014.01.012 24462697

[10] Oestereich L Lüdtke A Wurr S: Successful treatment of advanced Ebola virus infection with T-705 (favipiravir) in a small animal model. *Antiviral Res.* 2014;105:17–21. 10.1016/j.antiviral.2014.02.014 24583123

[11] Bishop BM: Potential and Emerging Treatment Options for Ebola Virus Disease. *Ann Pharmacother.* 2015;49(2):196–206. 10.1177/1060028014561227 25414384

[12] Qiu X Wong G Audet J: Reversion of advanced Ebola virus disease in nonhuman primates with ZMapp. *Nature.* 2014;514(7520):47–53. 10.1038/nature13777 25171469 PMC4214273

[13] Mupapa K Massamba M Kibadi K: Treatment of Ebola hemorrhagic fever with blood transfusions from convalescent patients. International Scientific and Technical Committee. *J Infect Dis.* 1999;179(Suppl 1):S18–23. 10.1086/514298 9988160

[14] Jahrling PB Geisbert JB Swearengen JR: Ebola hemorrhagic fever: evaluation of passive immunotherapy in nonhuman primates. *J Infect Dis.* 2007;196(Suppl 2):S400–3. 10.1086/520587 17940976

[15] Gulland A: First Ebola treatment is approved by WHO. *BMJ.* 2014;349:g5539. 10.1136/bmj.g5539 25200068

[16] Burnouf T Seghatchian J: Ebola virus convalescent blood products: where we are now and where we may need to go. *Transfus Apher Sci.* 2014;51(2):120–5. 10.1016/j.transci.2014.10.003 25457751 PMC7106377

[17] Gehring G Rohrmann K Atenchong N: The clinically approved drugs amiodarone, dronedarone and verapamil inhibit filovirus cell entry. *J Antimicrob Chemother.* 2014;69(8):2123–31. 10.1093/jac/dku091 24710028 PMC7110251

[18] Madrid PB Chopra S Manger ID: A systematic screen of FDA-approved drugs for inhibitors of biological threat agents. *PLoS One.* 2013;8(4):e60579. 10.1371/journal.pone.0060579 23577127 PMC3618516

[19] Nagata T Lefor AK Hasegawa M: Favipiravir: A New Medication for the Ebola Virus Disease Pandemic. *Disaster Med Public Health Prep.* 2014;1–3. 10.1017/dmp.2014.151 25544306

[20] Kouznetsova J Sun W Martínez-Romero C: Identification of 53 compounds that block Ebola virus-like particle entry via a repurposing screen of approved drugs. *Emerg Microbes Infect.* 2014;3:e84 10.1038/emi.2014.88 26038505 PMC4317638

[21] Turone F: Doctors trial amiodarone for Ebola in Sierra Leone. *BMJ.* 2014;349:g7198. 10.1136/bmj.g7198 25429872

[22] Wool-Lewis RJ Bates P: Characterization of Ebola virus entry by using pseudotyped viruses: identification of receptor-deficient cell lines. *J Virol.* 1998;72(4):3155–60. 9525641 10.1128/jvi.72.4.3155-3160.1998 PMC109772

[23] Howard W Hayman A Lackenby A: Development of a reverse genetics system enabling the rescue of recombinant avian influenza virus A/Turkey/England/50-92/91 (H5N1). *Avian Dis.* 2007;51(1 Suppl):393–5. 10.1637/1933-5334(2007)2[e46:TDOARG]2.0.CO;2 17494592

[24] Sandrin V Boson B Salmon P: Lentiviral vectors pseudotyped with a modified RD114 envelope glycoprotein show increased stability in sera and augmented transduction of primary lymphocytes and CD34 ^+^ cells derived from human and nonhuman primates. *Blood.* 2002;100(3):823–32. 10.1182/blood-2001-11-0042 12130492

[25] Salvador B Sexton NR Carrion R Jr: Filoviruses utilize glycosaminoglycans for their attachment to target cells. *J Virol.* 2013;87(6):3295–304. 10.1128/JVI.01621-12 23302881 PMC3592117

[26] Moncorgé O Mura M Barclay WS: Evidence for avian and human host cell factors that affect the activity of influenza virus polymerase. *J Virol.* 2010;84(19):9978–86. 10.1128/JVI.01134-10 20631125 PMC2937815

[27] Temperton NJ Hoschler K Major D: A sensitive retroviral pseudotype assay for influenza H5N1-neutralizing antibodies. *Influenza Other Respi Viruses.* 2007;1(3):105–12. 10.1111/j.1750-2659.2007.00016.x 19453415 PMC4941878

[28] Wright E Temperton NJ Marston DA: Investigating antibody neutralization of lyssaviruses using lentiviral pseudotypes: a cross-species comparison. *J Gen Virol.* 2008;89(Pt 9):2204–13. 10.1099/vir.0.2008/000349-0 18753230 PMC2886951

[29] Mather ST Wright E Scott SD: Lyophilisation of influenza, rabies and Marburg lentiviral pseudotype viruses for the development and distribution of a neutralisation -assay-based diagnostic kit. *J Virol Methods.* 2014;210C:51–8. 10.1016/j.jviromet.2014.09.021 25286181

[30] Chikte S Panchal N Warnes G: Use of LysoTracker dyes: a flow cytometric study of autophagy. *Cytometry A.* 2014;85(2):169–78. 10.1002/cyto.a.22312 23847175

[31] Dean RT Jessup W Roberts CR: Effects of exogenous amines on mammalian cells, with particular reference to membrane flow. *Biochem J.* 1984;217(1):27–40. 6365083 10.1042/bj2170027 PMC1153178

[32] Fellenius E Berglindh T Sachs G: Substituted benzimidazoles inhibit gastric acid secretion by blocking (H ^+^ + K ^+^)ATPase. *Nature.* 1981;290(5802):159–61. 10.1038/290159a0 6259537

[33] Elander B Fellenius E Leth R: Inhibitory action of omeprazole on acid formation in gastric glands and on H ^+^,K ^+^-ATPase isolated from human gastric mucosa. *Scand J Gastroenterol.* 1986;21(3):268–72. 10.3109/00365528609003075 3012768

[34] Lalezari JP Eron JJ Carlson M: A phase II clinical study of the long-term safety and antiviral activity of enfuvirtide-based antiretroviral therapy. *AIDS.* 2003;17(5):691–8. 12646792 10.1097/00002030-200303280-00007

[35] Freiberg AN Worthy MN Lee B: Combined chloroquine and ribavirin treatment does not prevent death in a hamster model of Nipah and Hendra virus infection. *J Gen Virol.* 2010;91(Pt 3):765–72. 10.1099/vir.0.017269-0 19889926 PMC2888097

[36] Ooi EE Chew JS Loh JP: *In vitro* inhibition of human influenza A virus replication by chloroquine. *Virol J.* 2006;3:39. 10.1186/1743-422X-3-39 16729896 PMC1481635

[37] Nuckols JT McAuley AJ Huang YJ: pH-Dependent entry of chikungunya virus fusion into mosquito cells. *Virol J.* 2014;11(1):215. 10.1186/s12985-014-0215-y 25476236 PMC4266220

[38] Pallister J Middleton D Crameri G: Chloroquine administration does not prevent Nipah virus infection and disease in ferrets. *J Virol.* 2009;83(22):11979–82. 10.1128/JVI.01847-09 19759137 PMC2772715

[39] De Lamballerie X Boisson V Reynier JC: On chikungunya acute infection and chloroquine treatment. *Vector Borne Zoonotic Dis.* 2008;8(6):837–9. 10.1089/vbz.2008.0049 18620511

[40] Tricou V Minh NN Van TP: A randomized controlled trial of chloroquine for the treatment of dengue in Vietnamese adults. *PLoS Negl Trop Dis.* 2010;4(8):e785. 10.1371/journal.pntd.0000785 20706626 PMC2919376

[41] Paton NI Lee L Xu Y: Chloroquine for influenza prevention: a randomised, double-blind, placebo controlled trial. *Lancet Infect Dis.* 2011;11(9):677–83. 10.1016/S1473-3099(11)70065-2 21550310

[42] Vigerust DJ McCullers JA: Chloroquine is effective against influenza A virus *in vitro* but not *in vivo*. *Influenza Other Respi Viruses.* 2007;1(5–6):189–92. 10.1111/j.1750-2659.2007.00027.x 19453426 PMC4941887

[43] Müller KH Kainov DE El Bakkouri K: The proton translocation domain of cellular vacuolar ATPase provides a target for the treatment of influenza A virus infections. *Br J Pharmacol.* 2011;164(2):344–57. 10.1111/j.1476-5381.2011.01346.x 21418188 PMC3174415

[44] Chandran K Sullivan NJ Felbor U: Endosomal proteolysis of the Ebola virus glycoprotein is necessary for infection. *Science.* 2005;308(5728):1643–5. 10.1126/science.1110656 15831716 PMC4797943

[45] Schornberg K Matsuyama S Kabsch K: Role of endosomal cathepsins in entry mediated by the Ebola virus glycoprotein. *J Virol.* 2006;80(8):4174–8. 10.1128/JVI.80.8.4174-4178.2006 16571833 PMC1440424

[46] Brecher M Schornberg KL Delos SE: Cathepsin cleavage potentiates the Ebola virus glycoprotein to undergo a subsequent fusion-relevant conformational change. *J Virol.* 2012;86(1):364–72. 10.1128/JVI.05708-11 22031933 PMC3255896

[47] Marzi A Reinheckel T Feldmann H: Cathepsin B & L are not required for ebola virus replication. *PLoS Negl Trop Dis.* 2012;6(12):e1923. 10.1371/journal.pntd.0001923 23236527 PMC3516577

[48] Gnirss K Kühl A Karsten C: Cathepsins B and L activate Ebola but not Marburg virus glycoproteins for efficient entry into cell lines and macrophages independent of TMPRSS2 expression. *Virology.* 2012;424(1):3–10. 10.1016/j.virol.2011.11.031 22222211 PMC7111950

[49] Ekins S Freundlich JS Coffee M: A common feature pharmacophore for FDA-approved drugs inhibiting the Ebola virus [v2; ref status: indexed, http://f1000r.es/4wt]. *F1000Res.* 2014;3:277 10.12688/f1000research.5741.2 25653841 PMC4304229

[50] Rutz M Metzger J Gellert T: Toll-like receptor 9 binds single-stranded CpG-DNA in a sequence- and pH-dependent manner. *Eur J Immunol.* 2004;34(9):2541–50. 10.1002/eji.200425218 15307186

[51] Hart OM Athie-Morales V O’Connor GM: TLR7/8-mediated activation of human NK cells results in accessory cell-dependent IFN-gamma production. *J Immunol.* 2005;175(3):1636–42. 10.4049/jimmunol.175.3.1636 16034103

[52] Shintani T Klionsky DJ: Autophagy in health and disease: a double-edged sword. *Science.* 2004;306(5698):990–5. 10.1126/science.1099993 15528435 PMC1705980

[53] White NJ Pukrittayakamee S Hien TT: Malaria. *Lancet.* 2014;383(9918):723–35. 10.1016/S0140-6736(13)60024-0 23953767

[54] Saliba KJ Kirk K: pH regulation in the intracellular malaria parasite, *Plasmodium falciparum*. H( ^+^) extrusion via a V-type H( ^+^)-ATPase. *J Biol Chem.* 1999;274(47):33213–9. 10.1074/jbc.274.47.33213 10559194

[55] Luciani F Spada M De Milito A: Effect of proton pump inhibitor pretreatment on resistance of solid tumors to cytotoxic drugs. *J Natl Cancer Inst.* 2004;96(22):1702–13. 10.1093/jnci/djh305 15547183

[56] De Milito A Fais S: Proton pump inhibitors may reduce tumour resistance. *Expert Opin Pharmacother.* 2005;6(7):1049–54. 10.1517/14656566.6.7.1049 15957961

[57] Perut F Avnet S Fotia C: V-ATPase as an effective therapeutic target for sarcomas. *Exp Cell Res.* 2014;320(1):21–32. 10.1016/j.yexcr.2013.10.011 24416789

[58] Avnet S Di Pompo G Lemma S: V-ATPase is a candidate therapeutic target for Ewing sarcoma. *Biochim Biophys Acta.* 2013;1832(8):1105–16. 10.1016/j.bbadis.2013.04.003 23579072

[59] De Milito A Iessi E Logozzi M: Proton pump inhibitors induce apoptosis of human B-cell tumors through a caspase-independent mechanism involving reactive oxygen species. *Cancer Res.* 2007;67(11):5408–17. 10.1158/0008-5472.CAN-06-4095 17545622

[60] Mizunashi K Furukawa Y Katano K: Effect of omeprazole, an inhibitor of H ^+^,K( ^+^)-ATPase, on bone resorption in humans. *Calcif Tissue Int.* 1993;53(1):21–5. 10.1007/BF01352010 8102318

[61] Tuukkanen J Väänänen HK: Omeprazole, a specific inhibitor of H ^+^-K ^+^-ATPase, inhibits bone resorption *in vitro*. *Calcif Tissue Int.* 1986;38(2):123–5. 10.1007/BF02556841 3006888

[62] Sheraly AR Lickorish D Sarraf F: Use of gastrointestinal proton pump inhibitors to regulate osteoclast-mediated resorption of calcium phosphate cements *in vivo*. *Curr Drug Deliv.* 2009;6(2):192–8. 10.2174/156720109787846225 19450226

[63] Yoshimura A Kuroda K Kawasaki K: Infectious cell entry mechanism of influenza virus. *J Virol.* 1982;43(1):284–93. 7109028 10.1128/jvi.43.1.284-293.1982 PMC256119

[64] Gasparini R Lai PL Casabona F: Do the omeprazole family compounds exert a protective effect against influenza-like illness? *BMC Infect Dis.* 2014;14:297. 10.1186/1471-2334-14-297 24889553 PMC4051147

[65] Lind T Rydberg L Kylebäck A: Esomeprazole provides improved acid control vs. omeprazole In patients with symptoms of gastro-oesophageal reflux disease. *Aliment Pharmacol Ther.* 2000;14(7):861–7. 10.1046/j.1365-2036.2000.00813.x 10886041

[66] Maitland K Williams TN Kotecka BM: Plasma chloroquine concentrations in young and older malaria patients treated with chloroquine. *Acta Trop.* 1997;66(3):155–61. 10.1016/S0001-706X(97)00046-6 9210966

[67] Long JS Wright E Molesti E: Inhibition of pseudotype virus entry by existing FDA-approved drugs. *Figshare.* 2014 Data Source

