# Supplementary material for: Antiviral therapies against Ebola and other emerging viral diseases using existing medicines that block virus entry
Source: F1000Res. 2015 Feb 10;4:30. Originally published 2015 Jan 29. [Version 2] doi: 10.12688/f1000research.6085.2 (PMC4431382; doi:10.12688/f1000research.6085.2)
Supplement: Peer review history

# Peer review: Referee response for version 2

**DOI:** 10.5256/f1000research.6568.r8175

**Published:** 2015-04-29

**Authors:** Robin A. Weiss

**Affiliations:** 1 Division of Infection and Immunity, University College London, London, UK

This is an elegant study employing envelope pseudotypes of highly pathogenic viruses which demonstrates that certain inhibitors of low endosomal pH can inhibit viral entry. Because some of these molecules such as Chloroquine have been in clinical use for decades, and are inexpensive, they might tip the balance between survival and death during human infection.

I have no criticism of the experimental work. However, I have been told by a reliable physician who has recently cared for patients with Ebola infection that treatment with Chloroquine offered no clinical benefit. Thus it is possible that an *in vitro *observation may not translate into a useful treatment *in vivo*. So one should be wary about the conclusions.

I have read this submission. I believe that I have an appropriate level of expertise to confirm that it is of an acceptable scientific standard.

# Peer review: Referee response for version 2

**DOI:** 10.5256/f1000research.6568.r8424

**Published:** 2015-04-27

**Authors:** Sean Ekins

**Affiliations:** 1 Collaborative Drug Discovery, Burlingame, CA, USA

The work was of particular interest especially in light of several viruses shown to be taken up in this rather non specific way. I would have perhaps also like some discussion of receptor and channel mediated virus uptake - there are several publications in this space. The interplay between such different mechanisms may point to multiple targets or need for combined approaches to block them.

The authors describe amiodarone, but there are many molecules that have been found as ebola replication or pseudoviral entry inhibitors, had they looked at more molecules to see if the pH mechanism was common across them?

I would likely suggest adding repurposing in the title of the article.

The conclusion might benefit from comparison of the chloroquine data with that previously published (higher EC50), potential ocular toxicity etc.

Some discussion as to whether the pH effect is an in vitro specific effect or something of in vivo relevance - would also be worth mention.

This study confirms the previous work on chloroquine and suggestions by others as to its potential utility. This begs the question why it is not used clinically. What other data would be needed to show that chloroquine could be clinically useful?

The study is well designed and reported and adds to the growing literature on chloroquine and its potential as an antiviral.

I have read this submission. I believe that I have an appropriate level of expertise to confirm that it is of an acceptable scientific standard.
